# Real-world outcomes of a dedicated fast-track polymyalgia rheumatica clinic

**DOI:** 10.1093/rheumatology/keae531

**Published:** 2024-09-30

**Authors:** Sharon Cowley, Patricia Harkins, Colm Kirby, Richard Conway, David Kane

**Affiliations:** Department of Rheumatology, Tallaght University Hospital, Dublin, Ireland; School of Medicine, Trinity College Dublin, Dublin, Ireland; School of Medicine, Trinity College Dublin, Dublin, Ireland; Department of Rheumatology, St James Hospital, Dublin, Ireland; Department of Rheumatology, Tallaght University Hospital, Dublin, Ireland; School of Medicine, Trinity College Dublin, Dublin, Ireland; School of Medicine, Trinity College Dublin, Dublin, Ireland; Department of Rheumatology, St James Hospital, Dublin, Ireland; Department of Rheumatology, Tallaght University Hospital, Dublin, Ireland; School of Medicine, Trinity College Dublin, Dublin, Ireland

**Keywords:** polymyalgia rheumatica, ultrasonography, delivery of health care, health policies, primary care rheumatology

## Abstract

**Objectives:**

To examine the clinical impact of a fast-track PMR clinic to enable early diagnosis and treatment, and to define both patient and disease characteristics in newly diagnosed PMR.

**Methods:**

Primary care physicians were invited to refer patients with new PMR to our fast-track clinic. Referral criteria included new onset shoulder or pelvic girdle pain and/or stiffness with elevated inflammatory markers in patients over 50 years. All patients were seen within 72 h of referral. Patients with a rheumatology diagnosis of PMR had an US of their temporal and axillary arteries.

**Results:**

172 patients were referred from primary care over 12 months. 39% of patients referred with suspected PMR had an alternative diagnosis for which PMR regimen glucocorticoids was inappropriate. 55% of the non-PMR diagnoses were other inflammatory rheumatological conditions requiring follow-up. Only 20% of patients referred from primary care already on glucocorticoids were commenced on bone protection. PMR patients were comorbid, with a mean of 2.5 other conditions. 75% of PMR patients experienced a glucocorticoid-related adverse event in the first 12 months of treatment. 17% of patients with new PMR had US features of subclinical GCA.

**Conclusion:**

The commencement of glucocorticoid therapy should be deferred until after specialist evaluation to enable an accurate clinical diagnosis. A delay in treatment can only realistically be avoided if general practitioners have access to a fast-track PMR clinic. We believe that rheumatologists should consider establishing fast-track PMR clinics and this study provides a strong case for and a template to support this practice innovation.

Rheumatology key messages

- PMR shares many symptoms with a wide range of inflammatory and non-inflammatory conditions.
- Specialist referral of PMR patients should be considered given potential diagnostic complexity and glucocorticoid side-effects.
- Deferring glucocorticoid treatment in suspected PMR until after specialist review may allow more accurate diagnosis.

## Introduction

PMR is one of the most common inflammatory rheumatic conditions in patients over 50 years, with a higher prevalence among women and in those of North European ethnicity [1]. The annual rate of incidence of PMR is estimated between 0.12 and 2.3 cases/1000 people aged over 50 years [2]. The incidence increases progressively with age, with a peak incidence occurring in those between 70 and 79 years of age [3]. The phenomenon of global population ageing will likely further contribute to an increased incidence over the coming decades. PMR is commonly characterized by pain and stiffness of the shoulders and hip girdle, but some patients may have features of peripheral arthritis or interspinal bursitis [4, 5]. Shoulder and hip girdle symptoms may also be the presenting feature of many other rheumatic and non-rheumatic conditions. Constitutional symptoms such as fever, fatigue, malaise and weight loss may be present which overlap with the symptoms of large vessel vasculitis and should prompt further evaluation of patients. To add to the complexity of presentation, it has been reported that almost a quarter of patients presenting with clinically isolated PMR have evidence of GCA on imaging studies [6] suggesting a disease continuum that has been recently described as GCA-PMR Spectrum Disease [7]. PMR is frequently accompanied by a rise in acute inflammatory markers including ESR and CRP concentrations, but this is not always the case. Consequently, it is essential to re-evaluate the concept of PMR, viewing it not as a monolithic clinical entity but rather as a complex, multifaceted clinical syndrome part of a larger spectrum of disease which includes GCA.

Despite being the most prevalent inflammatory rheumatic disease in those over the age of 50 years [8, 9], there is a distinct paucity of research published on PMR, compared with other rheumatological conditions. This is largely because in the majority of countries PMR is diagnosed and managed in primary care, with only a minority of cases referred for specialist evaluation [10]. However, over the past decade, there has been increased awareness of the diagnostic challenge that PMR presents, and of the potential morbidity and disability associated with both the disease, and the immunosuppression required for its management. The 2010 British Society of Rheumatology recommendations for the management of PMR advised specialist review of PMR patients with both atypical presentations and treatment-resistant disease/glucocorticoid contraindications [11]. The international GCA/PMR study group has recently advised that all patients with suspected or recently diagnosed PMR should be considered for specialist evaluation [12]. A Delphi exercise by PMR specialists included the recommendation for primary care to undertake a thorough history and clinical examination with basic laboratory investigations and for those with severe symptoms to be seen in a rapid access clinic, preferably with deferral of glucocorticoid therapy. Despite all this guidance, referral to secondary care only occurs in a minority of cases with an incomplete response or treatment-related adverse events. Few centres offer an early referral pathway, mainly due to capacity issues. Our objective was to assess the effectiveness and clinical impact of a newly established multicentre fast-track clinic to diagnose PMR. Our secondary objectives were to better define both patient and disease characteristics in those newly diagnosed with PMR in the community to inform future planning of rheumatology services.

## Methods

### Design and study population

This multicentre prospective longitudinal cohort study was conducted at the Rheumatology Departments in St James Hospital and Tallaght University Hospital, Dublin, Ireland. Primary care physicians in our catchment area with a population of 600 000 were invited to refer patients over 50 years with suspicion of new PMR to our fast-track clinic. Referral criteria included new-onset bilateral shoulder or pelvic girdle pain with elevated inflammatory markers and/or early morning stiffness. Primary care physicians were advised that patients should ideally be steroid naïve, or at least on steroid therapy for <1 month. Referrals were accepted via e-mail to a dedicated PMR clinic address, along with the patient’s history, laboratory results and contact details. The patients were contacted by telephone on the same working day with an appointment time arranged within 72 hours. All patients with a diagnosis of PMR on glucocorticoids for <4 weeks at the time of referral were followed up at 1-month, 3-month, 6-month and 12-month time points. All diagnoses of PMR were confirmed at 12 months.

### Fast-track PMR clinic

All patients referred to the fast-track clinic underwent a thorough history, clinical examination and extensive laboratory evaluation. A diagnosis of GCA, and other PMR mimics were actively sought. Baseline ESR, CRP, full blood count, liver profile and renal function were recorded. Additionally, each patient underwent ANCA, CTD, RF, anti-CCP, creatine kinase and extended myositis panel testing. Plain film radiographs of the shoulders were completed in all patients. The diagnosis of PMR was a clinical one, made in agreement by two rheumatologists. All patients diagnosed with PMR were assessed with vascular US of their temporal and axillary arteries.

US of all six branches of the superficial temporal arteries and both axillary arteries was performed using a GE P9 device. Sonographic abnormalities considered indicative of vasculitis in the temporal arteritis included the halo sign with a thickened, non-compressible intima-media complex. Cut-off values of ≥0.29 mm for the parietal branch, ≥0.34 mm for the frontal branch and ≥0.42 for the common temporal branch were used [13]. In the axillary arteries, the presence of a halo sign, and an intima-media thickness of >1.0 mm was considered positive. This has been shown to have 100% sensitivity and specificity in the common, frontal and axillary branches, and 97.2% sensitivity and 98.8% specificity in the case of the parietal branch [13]. A subset of patients with a clinical diagnosis of PMR had an US of their bilateral shoulder joints. These patients were selected at random and included both patients with isolated PMR and subclinical GCA in PMR.

If a diagnosis of new PMR was made, prednisolone was prescribed per the EULAR PMR guidelines [14] with a starting dose of 12.5–25 mg and tapered to a dose of 10 mg by 4–8 weeks, and by 1 mg per month once remission was achieved. Patients who did not strictly meet the referral criteria and who had been prescribed glucocorticoid treatment for >4 weeks at the time of referral were discharged back to primary care if they had a diagnosis of PMR with an appropriate glucocorticoid response. A plan was provided to primary care for glucocorticoid tapering. Patients with alternative diagnoses to PMR were either discharged or followed up in the general rheumatology clinic depending on the diagnosis.

### Ethical approval

Full ethical approval was obtained from the Joint Research Ethics Committee for Tallaght University Hospital and St James Hospital, Dublin, Ireland. All patients gave fully informed written consent to participate in the fast-track clinic study.

### Statistics

Descriptive analysis of the data was performed using STATA MP version 18. Data were expressed as the mean ± s.d. for normally distributed continuous variables, as median (interquartile range) for non-normally distributed variables, and as percentages and frequencies for categorical variables. Continuous variables were compared using the independent *t*-test. Categorical variables are summarised by frequency and percentage (%) and compared using the χ^2^ test or Fisher’s exact test. *P*-values <0.05 were considered statistically significant.

## Results

**Figure 1.**
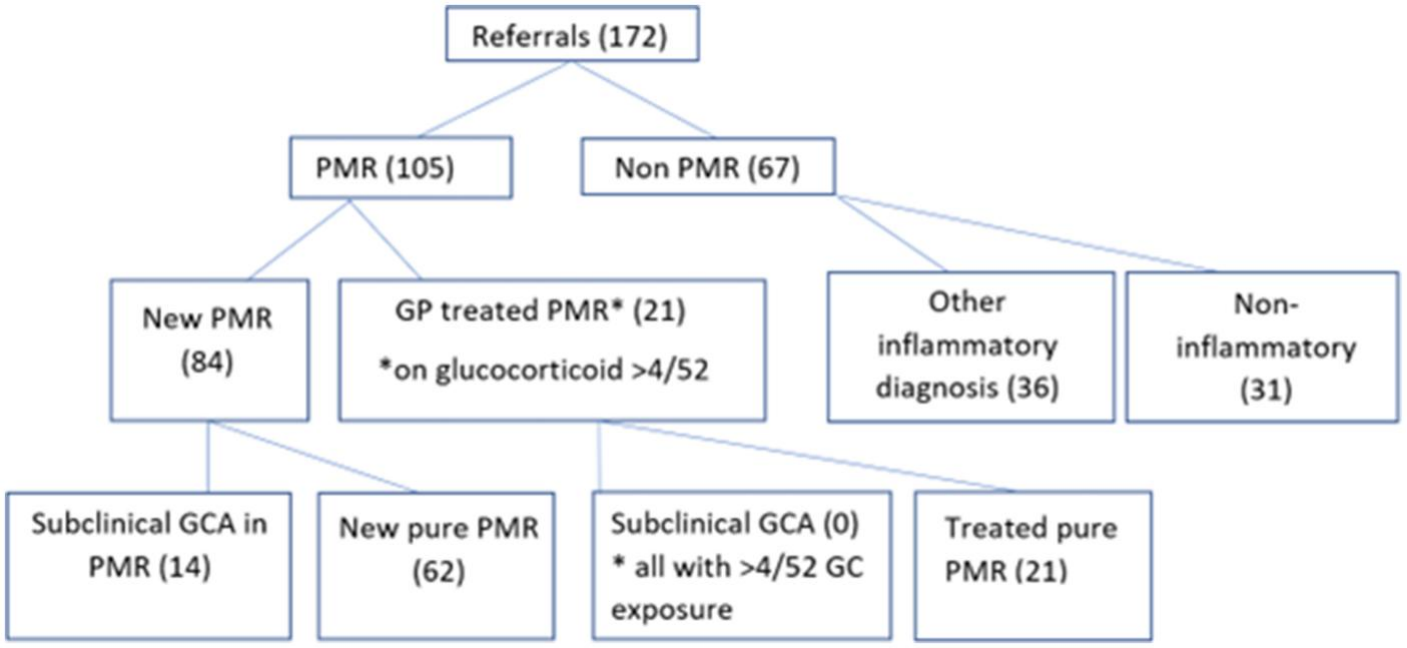
Referrals to fast-track PMR clinic

Between July 2022 and July 2023, a total of 172 patients were referred from primary care with new suspected PMR (see Fig. 1). This represented an average of 14 referrals a month or 2.4 referrals per 100 000 catchment population. Eighty-four (49%) patients met a clinical diagnosis of new PMR with agreement between two rheumatologists and were treated with a starting dose of glucocorticoid between 12.5 and 25 mg. Regarding presenting symptoms, 84 (100%) of patients with new PMR had bilateral shoulder pain, 74 (88%) had early morning stiffness, with the mean duration of stiffness lasting 164.19 min [±116.10], 71 (88%) reported fatigue, 59 (70%) had bilateral hip involvement, 25 (29%) reported anorexia, with 18 (21%) reporting weight loss. Twenty (24%) patients reported peripheral joint involvement, 15 (18%) had night sweats and 9 (11%) reported fevers. No patient was diagnosed with concomitant GCA on clinical assessment and no patient had cranial symptoms of headache, jaw claudication, scalp tenderness or visual disturbance. Fourteen of 84 patients diagnosed with PMR (17%) had US features of subclinical GCA at the time of diagnosis. A further 21 patients (12%) were already on corticosteroid treatment >10 mg for >4/52 weeks from primary care for suspected PMR without active features at the time of initial rheumatology review. None of these patients had US-identified subclinical GCA, likely due to their duration of glucocorticoid exposure. All of these patients had a good initial response to glucocorticoids and were discharged to primary care with a plan for a glucocorticoid taper over 6–8 months. These patients did not require re-referral to the service during their first year of treatment.

Sixty-seven patients (38.9%) had an alternative diagnosis to PMR with the majority having an alternative rheumatological diagnosis (see Table 1). The non-PMR diagnoses included seropositive inflammatory arthritis (4), seronegative inflammatory arthritis (17), inflammatory myopathy (4), RS3PE (1), crystal arthropathy (2), reactive arthritis (2), sarcoidosis (1), polyarteritis nodosa (1), ANCA vasculitis (4), OA (17), fibromyalgia (FM) (3), OA/FM overlap (9), and paraneoplastic (2) including ovarian cancer and prostate cancer. Some 57.3% of the non-PMR patients required at least one further rheumatology consultation.

**Table 1.** Alternative diagnoses from fast-track PMR clinic

Alternative diagnosis (total 67)	Number (*n*)
Inflammatory causes	
Seropositive inflammatory arthritis	4
• RF + only (3) • Anti-CCP + only (0) • RF and anti-CCP + (1)	
Seronegative inflammatory arthritis	17
Inflammatory myopathies	4
• PL7+ (1) • Ro52+ (1) • SRP+ (1) • Jo1+ (1)	
RS3PE	1
Crystal arthropathy	2
• Gout (1) • CPPD (1)	
Reactive arthritis	2
Sarcoidosis (confirmed on BAL)	1
Polyarteritis nodosa	1
ANCA vasculitis	4
• MPO + (3) • Atypical (1)	
Total inflammatory diagnoses	36
Other	
OA	17
FM syndrome	3
OA/FM overlap	9
Malignancy	2
• Ovarian cancer (1) • Prostate cancer (1)	
Total non-inflammatory diagnoses	31

### Patient characteristics

The mean age of this PMR cohort at diagnosis was 70.95 years [±7.95]. The baseline ESR in those with new PMR was 46.6 mm/h [±27.4] compared with 39.3 mm/h [± 27.1] in those with an alternative diagnosis to PMR (*P* = 0.11) (see Table 2). The mean baseline CRP in new PMR was 36.1 mg/l [±37.7] compared with 21.5 mg/l [±30.7] in those with an alternative diagnosis (*P* = 0.0018). Some 89.5% of those with a clinical diagnosis of new PMR made by two consultant rheumatologists met the 2012 ACR/EULAR PMR classification criteria [15]. In addition, 71% of patients with a non-PMR diagnosis also met the classification criteria (*P* = 0.010), highlighting caution against use as a diagnostic criterion. The median (interquartile range) number of days from symptom onset until PMR diagnosis in the fast-track clinic was 63.5 [30–95] days. The PMR cohort had a mean of 2.5 other comorbidities, with a median Elixhauser Comorbidity Index score of 0. The most common comorbidities affecting >25% of PMR patients were hypertension (58%), hyperlipidaemia (51%), OA (30%) and obesity (27%). Significantly 14% had type 2 diabetes at the time of PMR diagnosis. A substantial amount of PMR patients (74%) were either overweight or obese (see Table 3). There was also a considerable smoking history with 8% being active smokers and 45% ex-smokers.

**Table 2.** Patient characteristics PMR vs non-PMR diagnosis

Patient characteristic	New PMR (84)	Non-PMR (67)	
Age (mean ± s.d.)	70.95 (±7.95)	69 (±9.9)	*P* = 0.157
Female gender [*n* (%)]	43 (51.2)	41 (61.2)	*P* = 0.219
Baseline ESR (mm/h ± s.d.)	46.6 (±27.4)	39.3 (±27.1)	*P* = 0.11
Baseline CRP (mg/l ± s.d.)	36.1 (±37.7)	21.5 (±30.7)	*P* = <0.01
Meeting 2012 ACR/EULAR PMR classification criteria [*n* (%)]	75/84 (89.5)	47/67 (70.7)	*P* = <0.0001

**Table 3.** New PMR patient characteristics

Variable	Overall
*N*	84
Female [*n* (%))	43 (51.2%)
Mean age at diagnosis (years)	70.95 [±7.95]
Mean BMI at diagnosis	28.4 [±4.99]
Normal BMI [*n* (%)]	19 (22.6%)
Overweight [*n* (%)]	28 (33.3%)
Obese class 1 [*n* (%)]	26 (30.9%)
Obese class 2 [*n* (%)]	7 (8.3%)
Obese class 3 [*n* (%)]	1 (1.1%)
Underweight	3 (3.6%)
Ethnicity [*n* (%)]	
White	78 (96.3%)
Asian	1 (1.2%)
Black	2 (2.5%)

### Utility of a PMR fast-track clinic

In total, 48 (57%) patients were steroid naïve at diagnosis and had a mean starting prednisolone dose of 16.77 mg [±6.14]. This was in comparison with a mean starting steroid dose of 23.18 mg [±10.06] in those who were established on steroid therapy by their primary care physician for <30 days before referral, *P* = 0.54. The mean cumulative steroid dose in these patients upon referral was 457.73 mg [±293.6]. Interestingly, of the 39 patients who were established on steroid therapy for <4/52 prior to referral, only 8 (20%) were on some form of bone protection, including vitamin D, calcium and/or a bisphosphonate. Of those with a final diagnosis of PMR, 25 (29%) had been trialled on a non-steroidal anti-inflammatory medication in primary care and 13 (15%) were prescribed an opioid medication before referral. The mean time from symptom onset to referral to the fast-track clinic was 11.43 weeks [±9.26]. Glucocorticoid-related adverse events were assessed over 12 months following diagnosis. A total of 53/84 (63%) patients experienced sleep disturbance secondary to steroid therapy, and 3/84 (4%) received a new diagnosis of type 2 diabetes mellitus. Four of 84 (5%) reported mood disturbance during treatment. Thirteen of 84 (15%) received antibiotics for infection, two had an episode of shingles during their treatment course, five (6%) suffered an osteoporotic wedge compression fracture and one patient had a low-impact humeral shaft fracture. Additionally, one patient had a non-ST elevation myocardial infarction, and one patient had a transient ischaemic attack. Five patients were diagnosed with malignancy identified in the fast-track PMR clinic during their PMR treatment (two colorectal cancers, one prostate cancer, one lung cancer and one neuroendocrine tumour), indicating a possible paraneoplastic cause for their PMR.

### Ultrasound features

Vascular US was completed on all PMR patients. Some 17% of patients on glucocorticoid for <4 weeks before referral had US features of subclinical GCA at the time of diagnosis. The patients with subclinical GCA had a significant male predominance at 79% compared with 49% males in the PMR with normal vascular US group, *P* = 0.0388. The mean age of subjects with subclinical GCA in PMR was 70 years [±7.2], while those with pure PMR had a mean age of 71.1 years [±8.48], *P* = 0.65. Those with subclinical GCA in PMR had slightly more glucocorticoid exposure at 470.7 mg [±649.1] compared with those with isolated PMR at 457.7 [±293.6], *P* = 0.941; at the time of initial rheumatology review despite both groups having a similar time from symptom onset to rheumatology review at 11.43 weeks [±9.26] and 11.24 weeks [±11.91] for PMR and subclinical GCA in PMR, respectively. ESR was higher at baseline in those with subclinical GCA in PMR compared with their pure PMR counterparts at 54.9 and 44.4 mm/h, respectively, but this was not statistically significant, *P* = 0.20. There was no difference in baseline CRP levels between the two groups, *P* = 0.98.

Subacromial-subdeltoid bursitis is one of the most helpful US features for PMR diagnosis [16]. As a pilot study, a subset of 22 patients underwent bilateral shoulder US. These patients were selected randomly, and the cohort included both patients with isolated PMR (19) and subclinical GCA in PMR (3). 58% had findings of bilateral subacromial subdeltoid (SASD) bursitis, 33% had unilateral SASD bursitis and bilateral biceps tendon pathology was present in 21%. This suggests our PMR cohort had a similar spectrum of disease to previously published cohorts [17] but this finding is limited by the fact that not all patients were assessed.

## Discussion

There are few reports regarding fast-track PMR clinics in the literature to inform service provision. Using the referral criteria described in the methods and with a catchment population of 600 000 we received 14 referrals a month and diagnosed seven cases of PMR a month over a 12-month period, giving an annual PMR incidence of 0.14/1000 population. This is at the lower end of the reported incidence, with Northern European incidence reported as 0.33–1.13 per 1000 population [18]. This may represent an underestimate with less severe cases of PMR continuing to be managed in primary care. Given recent recommendations for early referral of cases of PMR for rheumatological assessment, these data are useful for planning services. Based on our figures, it is estimated that a rapid access PMR clinic would require 0.55 new patient slots per week per 100 000 catchment population and 1.17 review slots per week per 100 000. As many PMR patients can usually be discharged back to primary care with a management plan, the rapid access clinic can be sustained with this level of capacity. This assumes that PMR patients continue to be treated according to current practice with steroid therapy only. If biologic or other steroid-sparing therapy is instituted in future PMR management then a greater review of patient capacity would need to be provided.

Despite providing access to primary care to the new fast-track clinic within 72 hours, the median time from symptoms onset to review by a rheumatologist was 63.5 [30–95] days. A similar delay was reported in the Danish study by Frølund *et al.* at 53 [31–83] days [19]. A narrative review of PMR clinics also identified a wide-ranging time from symptom onset to specialist review of 24 days to 26 months [20], with fast-track strategies representing a much shorter time to rheumatology review. Reasons may include patient difficulty in accessing primary care, general practitioners (GPs) being unaware of the new fast-track clinic service or lack of awareness regarding the various presentations of PMR. One study that implemented a GP educational programme on PMR reduced the time from onset of symptoms to fast-track clinic referral from 42.9 [±15] to 24.3 [±12.5] days [21]. This may be a consideration to achieve a shorter time from symptom onset to fast-track review.

There was significant heterogeneity in the clinical presentations and final diagnosis of patients referred to the PMR clinic. Some 39% (67/172) of patients referred to the clinic with a primary care diagnosis of PMR had an alternative diagnosis as compared with 26% (29/112) patients in the study by Frølund *et al.* [19]. The lower rate of non-PMR in the Frølund study may reflect their strict referral requirement for an elevated CRP, while we accepted either elevated ESR or CRP and/or age over 50 years with a clinical presentation consistent with PMR. In both studies, the rate of cancer identified was low but not negligible. While the 2012 ACR/EULAR classification criteria for PMR did aid discrimination of PMR from non-PMR, 70% of non-PMR cases met the classification criteria. This highlights a limitation of simply using these criteria in routine clinical practice for early diagnosis without a thorough diagnostic work-up for other conditions. Most of the non-PMR patients (55%), had an alternative inflammatory rheumatological diagnosis for which PMR regimen glucocorticoid therapy was inappropriate. Osteoarthritis and seronegative inflammatory arthritis were the most common alternative diagnoses to PMR, but there were several patients with serious pathology including ANCA vasculitis, polyarteritis nodosa, myositis and co-existing new malignancy. These conditions are more challenging to diagnose in primary care with more limited consultation time for patient assessment and more limited access to diagnostics.

PMR exerts a profound impact on patients’ lives in multiple aspects including glucocorticoid-related adverse events, comorbidities and quality of life. Appropriate therapy with glucocorticoids rapidly reverses the morbidity and disability caused by PMR. However, 39% of patients with a community diagnosis of PMR were found to have an alternative diagnosis after rheumatologist review and treatment with PMR regimen glucocorticoids was inappropriate. Steroid-naïve PMR patients in our cohort also benefitted from lower starting glucocorticoid doses, than those prescribed in primary care (16.77 [±6.14] vs 23.18 [±10.06] mg). The Danish fast-track study also identified a higher starting glucocorticoid dose from GPs and other specialties compared with the doses prescribed in their fast-track clinic [19]. This is highly relevant as our PMR patients, had a high burden of glucocorticoid-related adverse events affecting over three-quarters of PMR patients. This included sleep disturbance, new type 2 diabetes, infections and new fractures. Given similar patient demographics and comorbidities, the non-PMR cohort would have likely experienced a similar rate of iatrogenic harm from glucocorticoids if the treatment had not been discontinued after their clinic assessment.

In addition to the glucocorticoid-related adverse events, a significant proportion of our PMR population already had a diagnosis of type 2 diabetes at the time of diagnosis (14%). The mean number of comorbidities in this cohort of 2.5 also reflected the propensity for glucocorticoid-related side effects such as loss of diabetic control and atherosclerosis in this older patient group. NSAIDs and opiates were prescribed for PMR-related pain in primary care before referral to the fast-track clinic in 29% and 15% of patients, respectively. In this older comorbid cohort, this is also not ideal due to the significant side effect profiles of these medications. Prior to referral to the PMR clinic, only 20% of patients initiated on glucocorticoid treatment were on some form of glucocorticoid-induced osteoporosis prophylaxis, including vitamin D, calcium and/or a bisphosphonate. This vulnerability of older PMR patients to all this potential iatrogenic harm can only be mitigated by establishing an early correct diagnosis such as is possible through a rapid access PMR clinic, appropriate use of glucocorticoid treatment and use of harm reduction strategies with consideration of glucocorticoid-sparing therapy.

The fast-track clinic provided an opportunity for vascular US scanning which allowed for risk stratification of these patients. The feasibility of implementing US in a fast-track PMR clinic depends on the resources of the national health systems and the regional health districts, but there is accumulating evidence that vascular US should be considered in the assessment of patients with PMR [22]. Musculoskeletal US was also an extremely useful adjunct for point-of-care diagnosis of rotator cuff disease and alternative shoulder pathology. It was also a valuable diagnostic aid in evaluating patients without acute-phase inflammatory rise, who may have been already started on glucocorticoids, when characteristic findings of PMR were present. It may also facilitate the administration of shoulder glucocorticoid injections which seem to be an effective and safe therapy for PMR [23].

A dedicated specialist PMR clinic significantly improved the diagnostic accuracy of PMR and ensured more appropriate treatment for the 39% of patients who did not have PMR. Older PMR patients are particularly vulnerable to significant iatrogenic harm and ‘low-dose’ PMR glucocorticoid therapy was associated with a significant level of side effects. The commencement of glucocorticoid therapy should ideally be deferred until after specialist evaluation to enable more accurate clinical diagnosis and prevent masking of laboratory and US parameters which may be useful for prognosis. However, a delay in therapy can only realistically be avoided if GPs have access to a fast-track PMR clinic. We believe that rheumatologists should consider establishing fast-track PMR clinics and this study provides a compelling case and a template to support this practice innovation.

## Data Availability

The data that support the findings of this study are available on request from the corresponding author (S.C.).
